# Injury mechanism affects the stability of suture-button syndesmosis fixation

**DOI:** 10.1186/s13018-020-02141-3

**Published:** 2020-12-10

**Authors:** Kuan-Hao Chen, Chih-Hwa Chen, Yu-min Huang, Hsieh-Hsing Lee, Yang-Hwei Tsuang

**Affiliations:** 1 Graduate Institute of Biomedical Materials and Tissue Engineering, College of Biomedical Engineering, Taipei Medical University, Taipei, Taiwan; 2 Department of Orthopedics, Shuang Ho Hospital, Taipei Medical University, New Taipei City, Taiwan; 3 Department of Orthopedics, School of Medicine, College of Medicine, Taipei Medical University, Taipei, Taiwan; 4 Department of Biomedical Engineering, National Taiwan University, Taipei, Taiwan; 5 School of Biomedical Engineering, College of Biomedical Engineering, Taipei Medical University, Taipei, Taiwan; 6 Research Center of Biomedical Device, Taipei Medical University, Taipei, Taiwan

**Keywords:** Syndesmosis injury, Suture-button, Diastasis, Dynamic fixation

## Abstract

**Background:**

Ankle syndesmosis injury is a common condition, and the injury mechanism can be sorted into pure syndesmosis injury, Weber-B, and Weber-C type fractures. This study aims to evaluate the treatment outcomes and stability of suture-button fixation for syndesmosis injury with different injury mechanisms. We hypothesized that injury mechanisms would alter the stability of suture-button fixation.

**Methods:**

We retrospectively reviewed 63 patients with ankle syndesmosis injury who underwent surgery with TightRope (Arthrex, Naples, FL, USA) from April 2014 to February 2019. The stability of suture-button fixation with TightRope was evaluated by comparing the preoperative, postoperative, and final follow-up measurements of tibiofibular clear space (TFCS), tibiofibular overlap (TFO), and medial clear space (MCS). A subgroup analysis for each demographic group and injury type including pure syndesmosis injury, Weber-B, and Weber-C type fractures were performed.

**Results:**

Syndesmosis was effectively reduced using TightRope. After the index surgery, the tibiofibular clear space was reduced from 7.73 to 4.04 mm, the tibiofibular overlap was increased from 3.05 to 6.44 mm, and the medial clear space was reduced from 8.12 to 3.54 mm. However, syndesmosis widening was noted at the final follow-up, especially in Weber-C type fractures (TFCS 3.82 to 4.45 mm, *p* < 0.01 and TFO 6.86 to 6.29 mm, *p* = 0.04). Though widened, the final follow-up values of tibiofibular clear space and tibiofibular overlap were in the acceptable range. Postoperatively and at the final follow-up, medial clear space was found to be significantly larger in the Weber-C group than in the pure syndesmosis and Weber-B groups (*p* < 0.05).

**Conclusions:**

Suture-button fixation can offer anatomic reduction and dynamic fixation in syndesmosis injuries. However, when using this modality for Weber-C type fractures, more attention should be focused on the accuracy of reduction, especially of medial clear space, and rediastasis should be carefully monitored.

**Trial registration:**

This trial was retrospectively approved by TMU-JIRB. Registration number N202004122, and the date of approval was May 06, 2020.

**Level of evidence:**

III

## Background

Ankle syndesmosis injury is a common condition, which can be a consequence of a simple fall, sports injury, motor vehicle accident, or fall from a height. The injury can occur as an isolated ligamentous tear or along with fractures. Studies have estimated that 10% of all ankle fractures and 20% of operatively treated ankle fractures occur along with syndesmosis injury [1, 2]. The pattern of ankle syndesmotic injuries may range from simple ligament sprains to diastasis of the syndesmosis involving structures and tissues around the ankle joint [3]. According to the classification system of ankle fractures created by Niels Lauge-Hansen, the mechanism of such injuries can be classified as supination-adduction, supination-external rotation, pronation-abduction, and pronation-external rotation [4–6]. Another classification system based on radiographic criteria created by Danis and Weber considers the relation between the distal fibular fractures and the syndesmosis [4, 7]. According to Danis-Weber classification, a type A fracture occurs distal to the syndesmosis, which correlates with supination-adduction injury by Lauge-Hansen [7]. A Weber type B fracture occurs at the level of syndesmosis, which correlates with supination-external rotation injury by Lauge-Hansen [7]. A Weber-C type fracture occurs above the level of syndesmosis, which correlates with pronation-abduction and pronation external rotation injury by Lauge-Hansen [7]. Weber-B type fracture and Weber-C type fracture may be related to syndesmosis injury [5, 7]. If syndesmosis injury is untreated, the initial 1 mm of the talus lateral shift may increase the tibiotalar contact pressure by 42%, which may result in ankle joint osteoarthritis [8]. Traditionally, transosseous screw fixation was used to fix the reduced syndesmosis. However, the treatment modality was associated with complications such as broken screws in 20.8% of patients and a requirement of secondary implant removal [9]. Another treatment option is suture-button fixation, which was introduced a decade ago [10]. The suture-button fixation was believed to maintain the fibular rotation during ankle motion when resisting diastasis [11]. Multiple prospective studies have proven that this novel fixation method is at least as effective as screw fixation, without a requirement of implant removal routinely [9, 11–13]. Alternatively, suture-button fixation was demonstrated to provide superior ankle dorsiflexion and plantar flexion [14]. Knot irritation (19%) and surgical site infection (8%) were believed to be the most common complications related to this technique [15–18]. The rate of failure of syndesmosis fixation by suture-button ranged from 2.8 to 4% [19–21]. Although abundant literature on syndesmosis fixation exists, up to our knowledge there is no study about the fixation stability based on the injury mechanism.

The present study evaluated the treatment radiologic outcomes and stability of fixation with suture-button fixation in syndesmosis injury with different injury mechanisms. We hypothesized that the stability of suture-button fixation would vary based on different injury mechanisms.

## Methods

In the present study, we retrospectively examined 63 patients who underwent suture-button fixation for ankle syndesmosis with TightRope (Arthrex, Naples, FL, USA) from January 2014 to February 2019. This study had 3 inclusion criteria adopted from previous studies: (1) presence of preoperative diastasis of tibiofibular clear space (TFCS) > 6 mm as measured in the standard anteroposterior (AP) view or mortise view, (2) preoperative MRI image with increased signal in T2-weighted imaging of interosseous space presenting disruption of the anteroinferior tibiofibular ligament (AITFL) or posteroinferior tibiofibular ligament (PITFL), and (3) occurrence of intraoperative movement > 3 mm after bony fixation by pulling of the fibula laterally with a towel clip [9]. Patients who meet either of the above criteria were included. Exclusion criteria were patients under 18 years old, occurrence or presence of an open fracture, inflammatory arthritis (rheumatoid or psoriatic), multiple fractures that prevented patients from immediate ambulation, and a concomitant head injury or neurologic deficit. The study was approved by Taipei Medical University—Joint Institutional Review Board.

### Surgical technique and rehabilitation

**Fig. 1:**
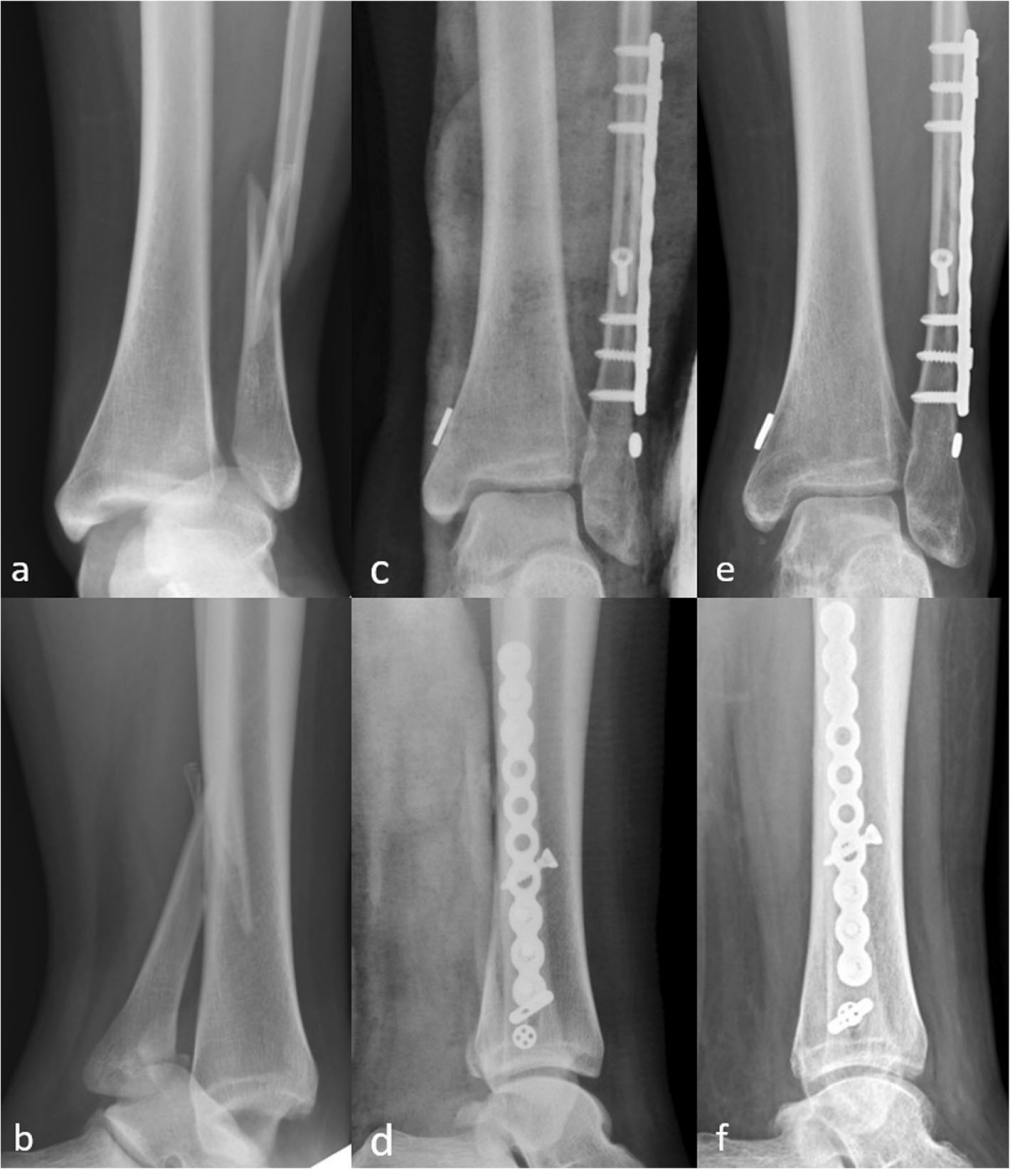
X-ray images of a man experiencing the Weber-C type ankle fracture with syndesmosis injury at 3 timepoints: preoperative (**a**, **b**), postoperative (**c**, **d**), and final follow-up (**e**, **f**)

Surgery was performed under general or spinal anesthesia in the supine position with a bump under the hip. If any fracture is present, open reduction and internal fixation with either plate or screws was done, followed by intraoperative evaluation of the syndesmosis by lateral pull of fibula. After syndesmosis instability was confirmed, syndesmosis fixation was performed through the following steps. First, open reduction of the syndesmosis was done under direct visualization and temporarily fixed with a k-wire. Thereafter, we used a guide pin to locate the optimal insertion site and trajectory. The site of insertion was 2–4 cm above the ankle joint aimed parallel to the joint and 20 to 30° anteriorly in the coronal plane from the fibula to tibia. After the guide pin was passed through the ideal tract, reaming was performed to open the canal for suture-button passage. The suture-button was passed through the guide needle with pull-through sutures, and then, the oblong button was flipped to rest on the medial cortex of the distal tibia. The position and reduction were determined under fluoroscopy, and then, the suture was tightened until the lateral button rested on the distal fibular cortex or lateral malleolar plate if present (Fig. 1).

After surgery, all patients were immobilized with a below-knee splint and maintained a non-weight-bearing status for 2 weeks and then protected weight bearing for another 4 weeks. Full weight bearing was allowed 6 weeks after surgery. A monthly outpatient follow-up was performed for at least 6 months. Implants were removed only if local discomfort was claimed or requested by the patient.

### Imaging methodology

Both AP, lateral, and mortise views of the ankle were taken at each time points, namely pre-operative, post-operative, and final follow-up. The pre-operative and post-operative X-rays were taken in supine position, and the final follow-up X-rays were taken under weight-bearing condition. The imaging techniques were standardized as follows.

For non-weight bearing AP views, the patient lay supine on examination table, with a pillow under head and leg fully extended. The foot was placed vertically on the image receptor, in neutral extended position. Slight foot pronation may be required for true AP position. For non-weight bearing mortise views, the patient positioned same as AP view, with the ankle internally rotated 15 to 20° until the intermalleolar line was parallel to the image receptor. For AP and mortise views, the central ray was perpendicular to the receptor and directed at a point midway between the malleoli [22, 23]. For non-weight-bearing lateral views, the patient lays in lateral recumbent position with the affected side down, flex the knee approximately 45°, and the opposite leg behind the injured leg. Place support under the knee as needed to place the leg and foot in a true lateral position, with the lateral malleolus about 1 cm posterior to the medial malleolus. Dorsiflex the foot so the plantar surface was at a right angle to the leg or as far as the patient could tolerate. The central ray was perpendicular to the receptor and directed to medial malleolus [22, 23].

For weight-bearing AP and lateral views, have the patient stand erect with weight evenly distributed on both feet. The feet should be directed straight ahead and parallel to each other. The placement of image receptor and direction of the central ray was same as non-weight-bearing views. For weight-bearing mortise views, have the patient stand erect with weight evenly distributed on both feet. Place the image receptor behind the feet, internally rotate the injured leg by 15 to 20°, until the intermalleolar line was parallel to the image receptor [22, 23].

For all above images, the source to image distance was set to 102 cm (40 in.) [22, 23].

**Fig. 2:**
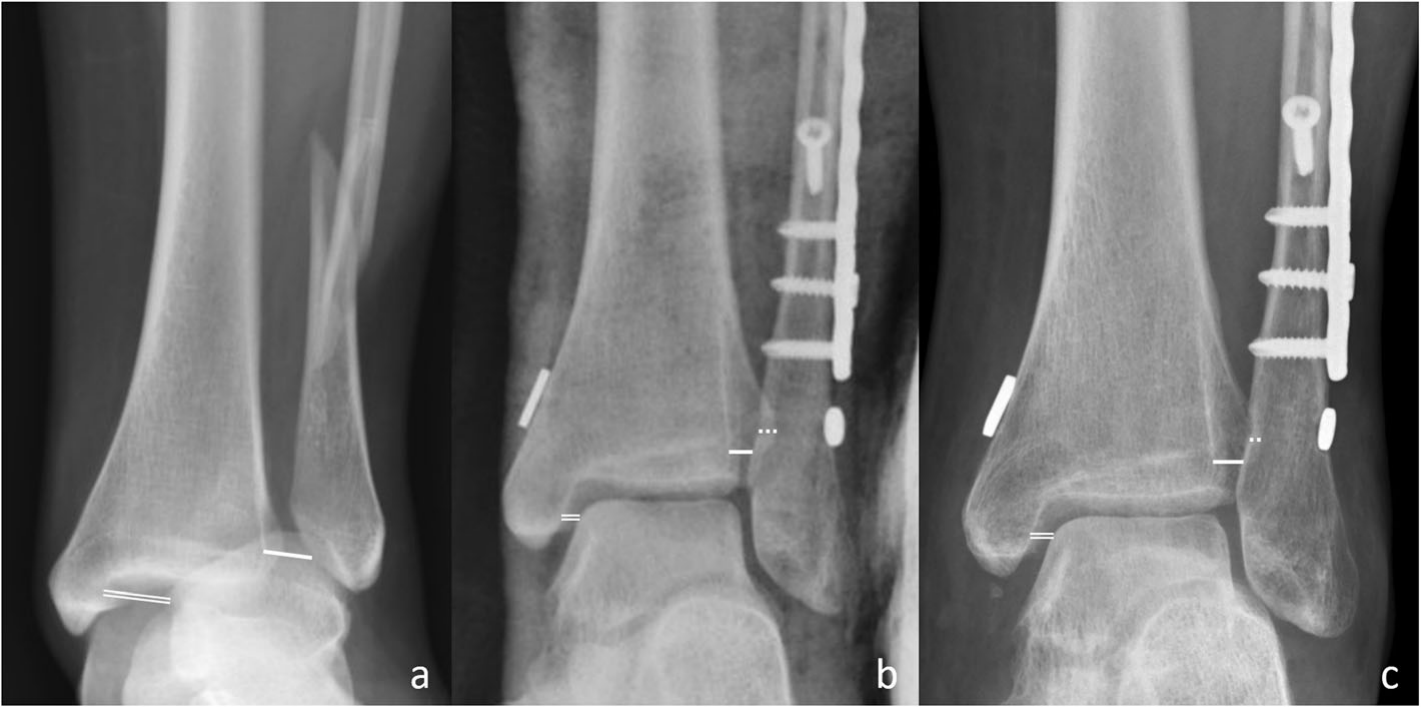
Exemplary X-ray images of the measurements commenced at different time points, preoperative (**a**), postoperative (**b**), final follow-up (**c**). The firm lines represent measurements of the tibiofibular clear spaces (TFCS), the double firm lines represent measurements of the medial clear spaces (MCS), and the dotted lines represent measurements of the tibiofibular overlaps (TFO)

Radiographic measurements were done at both 3 time points mentioned above. The tibiofibular clear space (TFCS) was measured as the distance between the medial border of the fibula and the incisura fibularis on a line parallel and 1 cm above the tibia plafond on AP view. The tibiofibular overlap (TFO) was measured as the maximum amount of overlap on mortise view [24, 25]. The medial clear space (MCS) was measured as the distance between the medial border of the talus and the lateral border of the medial malleolus on a line parallel and 5 mm below the talar dome [25, 26]. All measurements were recorded to the nearest 0.1 mm (Fig. 2).

### Statistical analysis

Patients were grouped based on the injury pattern, which included pure syndesmotic injury, Weber-B type fracture, and Weber-C type fracture [27]. The paired *t* test was used to compare preoperative, postoperative, and final follow-up measurements. Then, the one-way analysis of variance test and Welch test were used for comparing final follow-up measurements between the groups. All analyses were performed using SPSS, version 19.0 (SPSS, Chicago, IL, USA).

## Results

### Patient demographics

A total of 63 patients were included, of whom 37 (59%) were men and 26 (41%) were women, with a mean age 41.4 years (range 18–71 years). Ten (16%) patients had pure syndesmotic injury, 25 (40%) had Weber-B type fracture, and 28 (44%) had Weber-C type fracture (Table 1). All patients received single suture-button fixation for their syndesmotic instability. The mean follow-up period was 19.9 months, ranging from 14 to 36 months. At the end of the follow-up, no deep wound infection was noted. However, 14 (22%) patients underwent implant removal, and the mean time to removal was 15.2 months after the index surgery, ranging from 6 to 24 months (Table 1).

**Table 1:** Patient demographics and group characterization

	Total	Pure syndesmosis	Weber-B	Weber-C	***p*** value
**Number**	63 (100%)	10 (16%)	25 (40%)	28 (44%)	
**Male**	37 (59%)	5 (50%)	12 (48%)	20 (71%)	0.25^a^
**Female**	26 (41%)	5 (50%)	13 (52%)	8 (29%)	
**Age (year)**	41.4 (18~71)	38.4 (24~57)	43.6 (23~71)	40.5 (18~66)	0.83^b^
**Duration (months)**	19.9 (14~36)	19.3 (15~27)	18.7 (14~25)	21.1 (14~36)	0.17^b^
**Removal**	14 (22%)	1 (10%)	6 (24%)	7 (26%)	0.63^a^

Patients were grouped according to the injury mechanisms, namely pure syndesmotic injury, Weber-B type fracture, and Weber-C type fracture. There were no significant differences in sex, age, follow-up duration, and implant removal rate between groups

^a^Commenced with the Chi-squared test

^b^Commenced with ANOVA test

### Comparison among preoperative, postoperative, and final follow-up measurements

In the whole patient group, the mean preoperative values of TFCS, TFO, and MCS were 7.73 ± 0.60, 3.05 ± 0.36, and 8.12 ± 0.80 mm, respectively, and the mean immediate postoperative values were 4.04 ± 0.13, 6.44 ± 0.28, and 3.54 ± 0.10 mm, respectively. The above data indicate that after the index surgery, a significant reduction in both TFCS and MCS and an increase in TFO were observed. The mean final follow-up values of TFCS, TFO, and MCS were 4.43 ± 0.12, 6.12 ± 0.27, and 3.67 ± 0.10 mm, respectively. When final follow-up values were compared with postoperative values, a significant increase in TFCS and MCS and a significant decrease in TFO were observed (Table 2).

**Table 2:** Comparison between preoperative, postoperative, and follow-up measurements and stratified by groups with paired *t* test

	Pre-OP (mm)	Post-OP (mm)	Follow-up (mm)	***p***-value^b^
**Overall**				
**TFCS**	7.73 ± 0.60	**4.04 ± 0.13**^a^	**4.43 ± 0.12**^c^	**< 0.01**
**TFO**	3.05 ± 0.36	**6.44 ± 0.28**^a^	**6.12 ± 0.27**^c^	**0.03**
**MCS**	8.12 ± 0.80	**3.54 ± 0.10**^a^	**3.67 ± 0.10**^c^	**0.02**
**Pure syndesmotic**				
**TFCS**	6.08 ± 0.44	**4.33 ± 0.20**^a^	4.52 ± 0.16	0.06
**TFO**	4.65 ± 0.71	**6.56 ± 0.99**^a^	6.8 ± 0.87	0.24
**MCS**	3.81 ± 0.45	3.08 ± 0.14	3.16 ± 0.12	0.25
**Weber-B type**				
**TFCS**	6.73 ± 0.39	**4.17 ± 0.21**^a^	4.38 ± 0.17	0.08
**TFO**	2.7 ± 0.45	**5.95 ± 0.40**^a^	5.7 ± 0.41	0.09
**MCS**	7.02 ± 0.99	**3.32 ± 0.14**^a^	3.43 ± 0.11	0.08
**Weber-C type**				
**TFCS**	9.24 ± 1.26	**3.82 ± 0.20**^a^	**4.45 ± 0.20**^c^	**< 0.01**
**TFO**	2.82 ± 0.65	**6.86 ± 0.40**^a^	**6.29 ± 0.38**^c^	**0.04**
**MCS**	10.63 ± 1.42	**3.89 ± 0.18**^a^	4.07 ± 0.17	0.1

*TFCS* tibiofibular clear space, *TFO* tibiofibular overlap, *MCS* medial clear space, *Pre-OP* preoperative, *Post-OP* postoperative

^a^Significant difference compared to preoperative values

^b^Follow-up value compared to postoperative values

^c^Significant difference compared to postoperative values

**Fig. 3:**
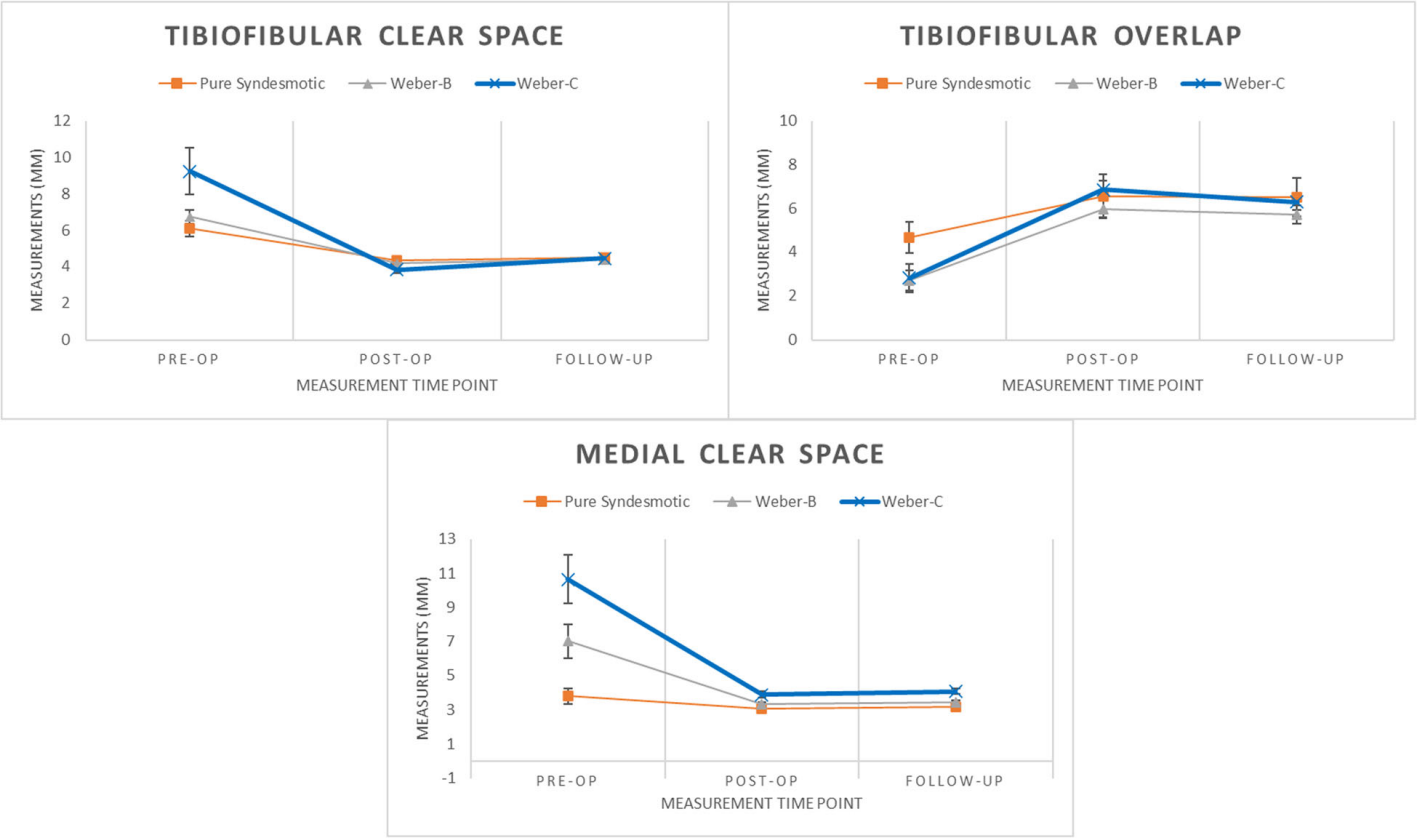
Trend for measurements at different time points. In Weber-C group, trend of increase in the tibiofibular clear space and trend of decrease in the tibiofibular overlap were found comparing immediate postoperative to final follow-up measurements (*p* < 0.05). The medial clear space in Weber-C group was wider than other groups at all time points (*p* < 0.05). Error bars stand as standard errors of the mean values

The same analysis was performed separately for each patient group, namely pure syndesmotic, Weber-B, and Weber-C. When postoperative and preoperative measurements were compared, we found a significant decrease in TFCS and MCS and a significant increase in TFO in each group. However, when the final follow-up and postoperative values were compared, the findings differ for each group. In the pure syndesmotic group, an increasing trend for TFCS and no significant change for MCS and TFO were observed. In the Weber-B group, increasing trends for TFCS and MCS and a decreasing trend for TFO were observed. Lastly, in the Weber-C group, significant increase in TFCS and significant decrease in TFO were observed (Table 2, Fig. 3).

### Comparison of final follow-up measurements between groups

To evaluate whether the trauma mechanism affected the stability of fixation, we first observed the number of patients with a widening of TFCS or MCS greater than 2 mm and a decrease of TFO greater than 2 mm. A TFCS change greater than 2 mm was observed in no (0%) patients in the pure syndesmosis group, 3 (11.1%) patients in the Weber-B group, and 4 (13.8%) patients in the Weber-C group. A TFO change greater than 2 mm was observed in no (0%) patients in the pure syndesmosis group, 1 (3.7%) patient in the Weber-B group, and 8 (27.6%) patients in the Weber-C group. Finally, an MCS change greater than 2 mm was observed in no (0%) patients at the final follow-up.

Regarding immediate post-surgery measurements, a significantly larger MCS was observed in the Weber-C group than in the pure syndesmosis (*p* = 0.003) and Weber-B groups (*p* = 0.029). Regarding final follow-up measurements, a significantly greater MCS was also observed in the Weber-C group than in the pure syndesmosis (*p* < 0.001) and Weber-B groups (*p* = 0.017). However, no significant difference was found between the groups for TFCS and TFO at both time points (Table 3).

**Table 3:** ANOVA for postoperative and follow-up measurements between groups

	***p*** value	Post hoc analysis^a^	***p*** value
**Post-operative**			
**TFC**	0.36		
**TFO**	0.38		
**MCS**	**0.006**^‡^	Pure syndesmotic ↔ Weber-B	0.51
		**Pure syndesmotic ↔ Weber-C**	**0.003**^‡^
		**Weber-B ↔ Weber-C**	**0.029**^‡^
**Follow-up**			
**TFC**	0.95		
**TFO**	0.31		
**MCS**	**0.001**^‡^	Pure syndesmotic ↔ Weber-B	0.17
		**Pure syndesmotic ↔ Weber-C**	**< 0.001**^‡^
		**Weber-B ↔ Weber-C**	**0.017**^‡^

*TFCS* tibiofibular clear space, *TFO* tibiofibular overlap, *MCS* medial clear space

^a^Commenced with Games-Howell test

^‡^*p* value < 0.05

## Discussion

In our study, suture-button fixation performed using the TightRope for ankle syndesmosis injury was found to be an effective modality, which resulted in a significant decrease in TFCS and MCS and a significant increase in TFO after the surgery, with acceptable alignment. However, postoperative measurements revealed that MCS was significantly larger in the Weber-C group than in the other 2 groups. Moreover, when final follow-up values were compared with postoperative values, we found a significant increase in TFCS and a decrease in TFO in the Weber-C group; however, the mean values of final follow-up TFCS and TFO were in the acceptable range.

In the Weber-C group, 12 patients (43%) had MCS greater than 4 mm after index surgery, 7 patients (25%) had TFO lesser than 6 mm, and 6 patients (21%) had TFCS greater than 6 mm. When postoperative measurements were compared between the groups, MCS was found to be significantly greater in the Weber-C group than in the other groups. The same results were also noted for final follow-up measurements, with the mean MCS value being slightly greater in the Weber-C group than in other groups.

To summarize the findings, the preoperative and immediate postoperative MCS were wider in Weber-C type fractures than in other types of injuries, and malreduction in MCS was more common in Weber-C type fractures when compared with different injury mechanisms. Alternatively, rediastasis was more likely to occur in Weber-C type fractures after weight bearing by the affected limb. These findings may be attributed to higher force applied to the affected ankle at the time of injury, greater initial displacement, and more commonly to fracture dislocation of the ankle. To date, various cohort studies have supported the use of suture-button fixation because it provides sufficient longevity and comparable outcomes as syndesmotic screw fixation [10, 12, 16, 18, 28–30]. However, Peterson et al. retrospectively reviewed 59 patients who underwent open reduction internal fixation of ankle syndesmosis with suture-button and found that the distance between the buttons increased with an average of 1.1 mm from immediate postoperative to final follow-up [21], which indicates widening of syndesmosis after weight bearing. However, the study did not focus on differences between injury mechanisms. In the study by Boden et al. [31] and Michelson and Waldman [32], the importance of deltoid ligament integrity on syndesmosis stability was emphasized. In the study, isolated sectioning of the interosseous ligament had no significant effect on syndesmotic stability, whereas sectioning of the deltoid ligament resulted in increased external rotation of the foot [2, 32]. Our results indicated that more deltoid ligament tears in Weber-C type fractures and more commonly malreduced MCS or inversed deltoid ligament after index surgery, causing instability of the syndesmosis. Thus, more rigid fixation may be needed for syndesmotic stability in Weber-C type fractures.

We also found widening of TFCS and MCS in nearly all groups after suture-button syndesmosis fixation. Andersen et al. compared difference of tibiofibular distance with the injured and uninjured leg, which found that the distance of the injured leg increased after 1 year and 2 years follow-up [11]. Teramoto et al. also demonstrated in a cadaveric study that diastasis of syndesmosis was more prominent with suture-button fixation than syndesmosis screw fixation [33]. Possible explanations for our results include overtightening of the suture-buttons, dynamic motions between tibia and fibula after weight bearing leading to osteolysis beneath the buttons, and initial malreduction of the syndesmosis. In this study, we focused on the radiological outcome of the patients. Further study might be needed to determine the threshold for acceptable syndesmosis widening after suture-button fixation.

The implant removal rate (22%) was higher in our study than in previous studies. Among the 14 patients who underwent implant removal, 7 underwent implant removal along with the removal of the lateral malleolar plate, whereas the remaining 7 underwent removal of the suture-button implants only. Furthermore, only 2 patients experienced severe soft tissue irritation and abscess formation. Knot irritation (19%) and surgical site infection (8%) were believed to be the most common complications related to this technique [15–18]. Naqvi et al. reported an implant removal rate of 16.7% with the standard technique and 0% with the modified technique of the suture-button. In the modified technique, 1 cm of the free ends of Fiberwire was buried in the recess behind the fibula and then was covered with the periosteum [16]. Storey et al. reported a similar technique to prevent skin irritation and abscess formation [17].

This study has several limitations. This retrospective study used only plain films for interpreting the button placement and syndesmosis distance and lacked a subjective functional score for outcome evaluation [33]. No verifications for reposition of the suture buttons by CT scans at immediate postoperative and last follow-up time points. Low numbers of our patient cohort, especially in the pure syndesmosis group, is also a major limitation of the study. Further large-scale prospective studies may be required for more definite interpretation of the current study results. A longer follow-up period is also required to observe the possibility of late diastasis and osteoarthritis.

## Conclusion

Suture-button fixation is an effective treatment modality for ankle syndesmosis injury combined with and without fracture. This technique leads to dynamic fixation without requiring implant removal routinely. However, when using this modality for Weber-C type fractures, more attention should be focused on the accuracy of reduction, especially of MCS, and rediastasis should be carefully monitored.

## Data Availability

The dataset analyzed during the current study are not publicly available due to patient’s privacy but are available from the corresponding author on reasonable request.

## Abbreviations

TFCS: Tibiofibular clear space
TFO: Tibiofibular overlap
MCS: Medial clear space
AITFL: Anteroinferior tibiofibular ligament
PITFL: Posteroinferior tibiofibular ligament
